# Robust Therapeutic Efficacy of Matrix Metalloproteinase-2-Cleavable Fas-1-RGD Peptide Complex in Chronic Inflammatory Arthritis

**DOI:** 10.1371/journal.pone.0164102

**Published:** 2016-10-14

**Authors:** Eon Jeong Nam, Jin Hee Kang, Keum Hee Sa, Shijin Sung, Jae Yong Park, Dong-Gyu Jo, Jae Hyung Park, In San Kim, Young Mo Kang

**Affiliations:** 1 Division of Rheumatology, Department of Internal Medicine, Kyungpook National University, School of Medicine, 680 Gukchaebosang-ro, Junggu, Daegu 41944, South Korea; 2 Cell and Matrix Research Institute, Kyungpook National University School of Medicine, 680 Gukchaebosang-ro, Junggu, Daegu 41944, South Korea; 3 Division of Pulmonology, Department of Internal Medicine, Kyungpook National University, School of Medicine, 680 Gukchaebosang-ro, Junggu, Daegu 41944, South Korea; 4 School of Pharmacy, Sungkyunkwan University, 2066 Seobu-ro, Jangangu, Suwon 16419, Republic of Korea; 5 Department of Polymer Science and Engineering, College of Engineering, Sungkyunkwan University, 2066 Seobu-ro, Jangangu, Suwon 16419, Republic of Korea; 6 Biomedical Research Institute, Korea Institute of Science and Technology, 5, Hwarang-ro 14-gil, Seongukgu, Seoul 02792, Republic of Korea; 7 Department of Biochemistry and Cellular Biology, Kyungpook National University School of Medicine, 680 Gukchaebosang-ro, Junggu, Daegu 41944, Republic of Korea; Universite de Nantes, FRANCE

## Abstract

**Objective:**

Therapeutic agents that are transformable via introducing cleavable linkage by locally enriched MMP-2 within inflamed synovium would enhance therapeutic efficacy on chronic inflammatory arthritis. Transforming growth factor-β-inducible gene-h3 (βig-h3), which consists of four fas-1 domains and an Arg-Gly-Asp (RGD) motif, intensifies inflammatory processes by facilitating adhesion and migration of fibroblast-like synoviocyte in the pathogenesis of rheumatoid arthritis (RA). The aim of this study was to investigate whether a MMP-2-cleavable peptide complex consisting of a fas-1 domain and an RGD peptide blocks the interaction between βig-h3 and resident cells and leads to the amelioration of inflammatory arthritis.

**Methods:**

We designed βig-h3-derivatives, including the fourth fas-1 domain truncated for H1 and H2 sequences of mouse (MFK00) and MMP-2-cleavable peptide complex (MFK902). MMP-2 selectivity was examined by treatment with a series of proteases. MFK902 efficacy was determined by the adhesion and migration assay with NIH3T3 cells *in vitro* and collagen-induced arthritis (CIA) model using male DBA/1J mice *in vivo*. The mice were treated intraperitoneally with MFK902 at different dosages.

**Results:**

MFK902 was specifically cleaved by active MMP-2 in a concentration-dependent manner, and βig-h3-mediated adhesion and migration were more effectively inhibited by MFK902, compared with RGD or MFK00 peptides. The arthritis activity of murine CIA, measured by clinical arthritis index and incidence of arthritic paws, was significantly ameliorated after treatment with all dosages of MFK902 (1, 10, and 30 mg/kg). MFK902 ameliorated histopathologic deterioration and reduced the expression of inflammatory mediators simultaneously with improvement of clinical features. In addition, a favorable safety profile of MFK902 was demonstrated *in vivo*.

**Conclusion:**

The present study revealed that MMP-2-cleavable peptide complex based on βig-h3 structure is a potent and safe therapeutic agent for chronic inflammatory arthritis, thus providing reliable evidence for a MMP-2-cleavable mechanism as a tissue-targeted strategy for treatment of RA.

## Introduction

Rheumatoid arthritis (RA) is a destructive autoimmune arthritis, in which inflammatory cell infiltration, synoviocyte proliferation, and augmented expression of matrix-degrading enzymes, such as matrix metalloproteinases (MMPs) and extracellular matrix (ECM) proteins, are critically involved in the development of synovial inflammation [1–5]. Transforming growth factor-β-inducible gene-h3 (βig-h3) is an ECM protein that consists of integrin-interacting structures, including four homologous fas-1 domains and an Arg-Gly-Asp (RGD) motif [6, 7], and is abundantly found within synovial tissues and fluid of RA [6, 8]. In the pathogenesis of RA, βig-h3 intensifies the inflammatory processes by facilitating adhesion and migration of fibroblast-like synoviocytes (FLS) via binding to αvβ3 integrin, which contributes significantly to the initiation and perpetuation of synovial pathology [6]. Furthermore, endothelial cell functions and pathologic angiogenesis, which are fundamental components of pannus development in RA [9, 10], are modulated by βig-h3 [7, 11]. In another inflammatory condition, vascular proinflammatory responses, including vascular permeability and leukocyte diapedesis by binding to the αvβ5 integrin, are related to the functions of βig-h3 [12]. Based on these functions, fragmented peptides of and an antibody against βig-h3 were developed as therapeutic agents for chronic inflammatory arthritis [13], sepsis [14], and tumours [15]. Although promising results have been documented for these strategies, more potent therapeutic agents are possibly required to be developed based on βig-h3.

MMPs are zinc-containing endopeptidases that play a key role during inflammatory responses [3, 4]. In rheumatoid synovium, MMPs are overexpressed and involved in excessive degradation of the ECM [1, 5]. Abundant MMPs in the inflamed synovial tissues may provide promising targets that can be utilized for prodrug activation [16]. We developed a novel MMP-1-cleavable composite peptide consisted of two different βig-h3 fragments, which showed markedly improved therapeutic efficacy in chronic inflammatory arthritis [13]. This proof-of-concept paved the way to the development of composite peptide prodrugs that can be activated by enzymes that are specifically enriched within the extracellular space of inflammatory arthritis. Therefore, exploitation of abundant MMPs in the microenvironment of rheumatoid synovium is another technical expansion of this proof-of-concept development of RA therapeutics.

Among MMPs, MMP-2 is associated with radiographic erosions in early RA [17]. MMP-2 expression, especially in its proteolytically active form, is highly upregulated [1, 18] within the invasive pannus at the junction of cartilage and bone [18, 19]. ProMMP-2 is recruited to the cell surface and activated partially by forming a cell membrane-associated ternary complex consisting of MT1-MMP/TIMP-2/MMP-2. Partially activated MMP-2 undergoes enzymatic maturation through binding to αvβ3 integrin by autocatalytic mechanisms [20–22]. These mechanisms induce localization of active MMP-2 to the cell surface, concentrating MMP-2 within the vicinity of its targets [18, 19, 23]. Furthermore, polarized colocalization of MMP-2 and αvβ3 integrin on the podosome-like structure during directional cell migration has been recognized as one of major contributor to the proteolytic degradation of ECM [22, 24, 25]. Therefore, therapeutic agents that are transformable via introducing a cleavable linkage targeted by cell surface-enriched MMP-2 within the pannus tissue would enhance targetability and therapeutic efficacy.

The present study investigated whether a MMP-2-cleavable peptide complex consisting of a fas-1 domain and an RGD motif blocks interactions between βig-h3 and resident effector cells, leading to the amelioration of inflammatory arthritis. The anti-inflammatory activity of this peptide complex was validated by measuring the inhibition of βig-h3-mediated cellular functions and monitoring therapeutic efficacy in an experimental arthritis model.

## Materials and Methods

### Materials

The reagents and their sources were as follows: bovine type II collagen, complete Freund’s adjuvant (CFA), and incomplete Freund’s adjuvant (IFA) were purchased from Chondrex (Redmond, WA). Recombinant human MMP-1, MMP-2, and MMP-3 were obtained from Chemicon (Temecula, CA), and recombinant human cathepsin D, cathepsin L, and cathepsin K were obtained from Calbiochem (La Jolla, CA). Polyclonal antibody was generated by immunizing rabbits with recombinant βig-h3. Antibodies against murine CD3 (Dako, Glostrup, Denmark), CD31 (clone MEC13.3; BD Bioscience, San Jose, CA), ICAM-1 (clone 166623) and receptor activator of nuclear factor kappa-B ligand (RANKL; clone 88227; R&D Systems, Minneapolis, MN), GAPDH (clone 6C5; Abcam, Cambridge, UK), human MMP-2 (Millipore, Billerica, MA), and the histidine-tag (Penta·His, Qiagen, Hilden, Germany) were used as primary antibodies. Secondary antibodies included horseradish peroxidase (HRP)-conjugated goat anti-rat and rabbit anti-goat, and biotinylated rabbit anti-rat and swine anti-rabbit antibodies (Dako).

### Production of Recombinant Peptides Based on βig-H3 Structure

RGD (GGRGDSP) and Asp-Gly-Glu (RGE; GGRGESP) peptides were synthesized by AnyGen (Gwangju, Republic of Korea). Complementary DNA (cDNA) of the 4th fas-1 truncated for H1 and H2 sequences of murine βig-h3 (designated MFK00) were generated by polymerase chain reaction (PCR) and cloned into the *EcoR*V and *Xho*I sites of the pET-29b(+) plasmid (Novagen, Madison, WI). In order to produce a peptide complex consisting of MFK00 and RGD linked by the MMP-2 substrate (Gly-Pro-Leu-Gly-Val-Arg-Gly; GPLGVRG), cDNA of the RGD peptide and GPLGVRG was cloned into the *EcoR*I, *Sal*I, and XhoI sites of the pET-29b(+) plasmid. A His-tag was attached to the RGD peptide to allow purification of the peptide complex by affinity chromatography. Endotoxin was removed using Triton X-114 in lysis buffer, followed by application of the EndoTrap Blue endotoxin removal system (Cambrex, Walkersville, MD). Lipopolysaccharide was tested using a QCL-1000 Chromogenic Endpoint LAL kit (Cambrex), and the concentration was maintained at 0.2–0.4 EU/mL (< 0.1 EU/mouse).

### Enzymatic Cleavage Assay

MMP-2-specific peptide cleavage was evaluated *in vitro* using proteases. MFK902 (3 μg/mL) was dissolved in an assay buffer (150 mM NaCl, 20 mM Tris, and 5 mM CaCl2, pH 7.5), followed by incubation with different concentrations of MMPs (MMP-1, -2, or -3) or cathepsins (cathepsin D, L, or K) at 37°C for 3 h [26]. To confirm cleavage of the MFK902, immunoblot assays using anti-His-tag and anti-βig-h3 antibodies were performed after separation of protein from media. Integrated intensity was measured for uncleaved MFK902, which was detected by the anti-βig-h3 antibody, and its cleaved form, which was detected by the anti-His-tag antibody, using NIS Elements Advanced Research version 3.2 software (Nikon Instruments, Tokyo, Japan). Data were generated by calculating the normalized ratios of cleaved and uncleaved MFK902 intensities.

### Cell Culture

NIH3T3 cells (CRL-1658), a murine fibroblast line, were obtained from ATCC (Manassas, VA) and cultured in Dulbecco's modified Eagle's medium containing 4.5 gm/L glucose supplemented with 100 U/mL penicillin, 100 μg/mL streptomycin, and 10% foetal calf serum at 37°C in an atmosphere of 5% CO_2_. Upon reaching confluence, cells were divided using trypsin/EDTA for detachment.

### Adhesion Assay

Cell adhesion assay was performed as described previously [6]. Briefly, murine recombinant wild-type βig-h3 proteins (0.5 μg/well) were coated in 96-well non-tissue culture plates (Costar, Cambridge, MA) for 16 h at 4°C. NIH3T3 cells (5 × 10^5^/mL) were added to coated wells and allowed to adhere at 37°C for 2 h. For the inhibition assay, RGD or βig-h3-derived peptides were preincubated with cells at 37°C for 1 h. Adherent cells were lysed by addition of 0.5% Triton X-100, followed by development with 3.75 mM *p*-nitrophenyl-*N*-acetyl β-D-glucosaminide in citrate buffer. The absorbance was measured at 405 nm in a microplate reader (Molecular Devices, Sunnyvale, CA).

### Migration Assay

The transwell plate with 8-μm pore size (Costar) was settled in a 24-well culture plate (Costar) and the bottom surface of the membrane was coated with recombinant murine wild-type βig-h3 proteins (2 μg/well) and incubated at 4°C for 16 h. NIH3T3 cells (1 × 10^6^/mL) were added to the upper chamber. For the inhibition assay, βig-h3-derived peptides were preincubated with cells at 37°C for 1 h. After allowing migration at 37°C for 7 h, cells were fixed with 4% paraformaldehyde and stained with crystal violet. The number of migrated cells was determined at nine random high-power fields using light microscopy.

### Animals

All animal care and experimental procedures were approved by the Kyungpook National University Institutional Animal Care and Use Committee (Approval Number: KNU 2014–46) and conducted in accordance with the institutional protocol for animal welfare. All mice were male DBA/1J strain between the ages of 8 and 10 weeks and maintained in an animal facility under specific pathogen-free conditions and a temperature-controlled environment. Standard chow and water were provided *ad libitum*. Mice were monitored three times per week. The development of arthritis in the peripheral joints was checked by two independent observers. The changes of body weights of mice were recorded at days 21, 35 and 50. After completion of the study, animals were anesthetized with isoflurane and sacrificed with CO_2_ inhalation. In cases of infection, unacceptable inflammation on the tail where type II collagen with either complete or incomplete Freund adjuvant is intradermally injected, or other serious conditions, animals were euthanized to avoid unacceptable distress or pain according to guideline from IACUC. Blood was collected by left cardioventricular puncture and centrifuged to obtain plasma and serum. Complete blood cell counts and serum chemistry assessments were performed to test hepatic and renal functions. Paw tissues were washed with phosphate-buffered saline (PBS) and snap frozen in liquid nitrogen for analytical studies. For histological assessment, the hind paws were removed and fixed in 10% neutral-buffered formalin. All studies involving animals are reported in accordance with the ARRIVE guidelines for reporting experiments involving animals [27, 28].

### In vivo optical imaging

Mice were classified into 2 groups—a control group that received free Cy5.5 and a second group that received MFK902-Cy5.5. Before imaging, mice were anesthetized via isoflurane inhalation, and 5 mg/kg of probe was administered to each mouse intravenously. After injections, fluorescence images were captured using an eXplore Optix system (Advanced Research Technologies Inc., Montreal, Canada). For each mouse, the total fluorescence intensity (TPC) was calculated over the 4 paws using the region of interest (ROI) function of the Analysis Workstation software (Advanced Research Technologies Inc) in order to assess the overall degree of arthritis.

### Induction and Clinical Assessment of Collagen-Induced Arthritis (CIA)

A murine CIA model was established as previously described with minor modifications [29]. This model of arthritis in mice has been in use for over 30 years [30, 31]. To induce CIA, DBA/1 mice were injected intradermally at the base of the tail with 100 μg of bovine type-II collagen (CII) emulsified in CFA (1:1 w/v) and boosted at day 21 with 100 μg of CII emulsified in IFA (1:1 w/v). The mice were randomized into 4 groups to receive either PBS or three dosages (1, 10, or 30 mg/kg) of MFK902 intraperitoneally. All mice continued MFK902 treatment at the same dose and route of administration as at entry. Clinical arthritis index (CAI) was scored using a previously described scale: 0, no arthritis; 1, one inflamed digit; 2, two inflamed digits; 3, more than two inflamed digits and foot-pad inflammation; 4, all digits inflamed. Each limb was graded with a score of 0–4, with a maximum possible score of 16 for each mouse. The incidence of arthritic paws was defined as the incidence of inflamed paws with a clinical arthritis score ≥ 2 among four paws.

### Histology and Immunohistochemistry

The hind paws were harvested on day 50, fixed in 10% neutral-buffered formalin for 48 h, decalcified in 10% EDTA at 4°C for 30 days, and embedded in paraffin. Sections of the joints were stained with haematoxylin and eosin (H&E) and scored using an arbitrary scale of 0 to 3 (0, normal; 1, mild; 2, moderate; 3, severe) for the following categories: synovial hyperplasia, pannus formation, cartilage destruction, and bone erosion [32]. For immunohistochemical analysis, antigen retrieval was performed at 60°C overnight in EDTA buffer (pH 9.0) for CD3 or with trypsin digestion for CD31. Endogenous peroxidase activity was depleted by treatment with 0.3% H_2_O_2_, and nonspecific binding was blocked with 5% bovine serum albumin. The sections were incubated with primary antibodies at appropriate dilutions, followed by the biotinylated secondary antibodies and Vectastain Elite ABC reagents (Vector Laboratories, Burlingame, CA).

### Semi-quantitative Reverse Transcription PCR (RT-PCR)

Total RNA was extracted from synovial tissues using an RNeasy extraction kit (Intron Biotech, Sungnam, Korea) and reverse transcribed to cDNA templates using the PrimeScript 1st-strand cDNA synthesis kit (Takara Bio, Shiga, Japan). For semi-quantitative PCR, cDNA was amplified using primers, Taqman probes, and a LightCycler 480 system (Roche, Basel, Switzerland) according to manufacturer recommendations. Gene transcript levels were calculated as target gene/reference gene (18S ribosomal RNA) ratios and normalized according to the relative expression level of non-arthritic mice.

### Immunoblot Analysis

For immunoblot analysis, the hind paws were homogenized and sonicated in the lysis buffer, followed by centrifugation at 15,000*g* at 4°C for 10 min. Proteins were fractionated by 15% sodium dodecyl sulphate-polyacrylamide gel electrophoresis and transferred to the nitrocellulose membranes using a semi-dry transfer system (Transblot; Bio-Rad). The membranes were blocked with 10% nonfat dry milk and incubated with specific primary antibodies, followed by HRP-conjugated secondary antibodies. The bands were visualized using ECL Plus chemiluminescence reagents (Amersham, Oakville, ON, Canada) and quantified by ImageJ software (National Institutes of Health, Bethesda, MD).

### Statistical Analysis

The data and statistical analysis comply with the recommendations on experimental design and analysis in pharmacology [33]. All quantitative values were expressed as the mean ± SEM. The statistical significance of differences between the treatment and control groups was analysed by Student’s *t* test or one-way analysis of variance (ANOVA), followed by Tukey’s post hoc test. Repeated-measures ANOVA with Tukey’s post hoc test were performed to compare differences between treatment groups at multiple time points. A *p <* 0.05 was considered to be statistically significant. Statistical analysis was performed using SPSS 12.0 software (SPSS Inc., Chicago, IL).

## Results

### Characterization of the MMP-2-cleavable Peptide Complex

**Fig 1:**
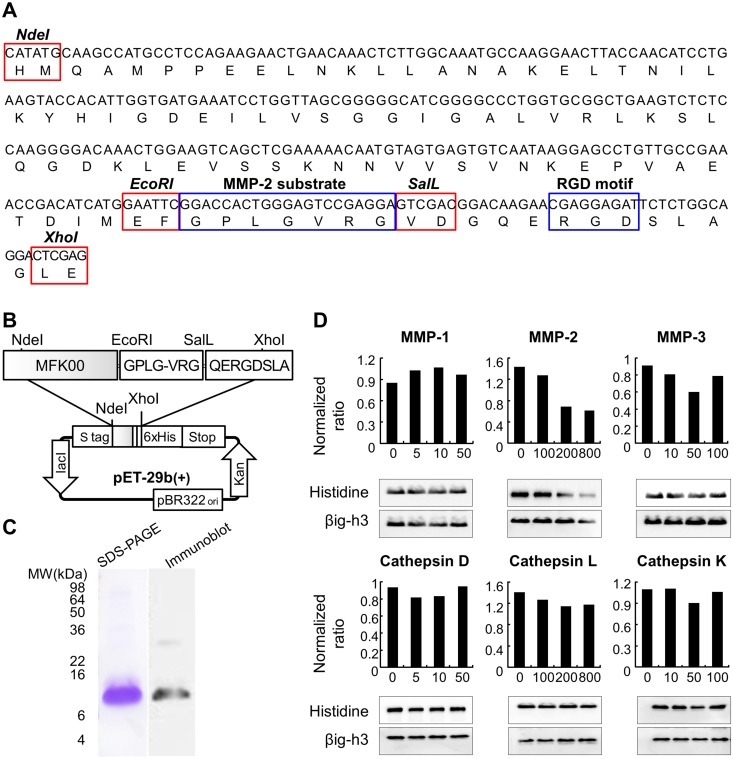
Identification of matrix metalloproteinase-2 (MMP-2)-specific cleavage of MMP-2-cleavable peptide complex, MFK902. (A) Sequence of recombinant murine MFK902 consisting of MFK00 (murine 4^th^ fas-1 peptide truncated for the H1 and H2 sequences) and an RGD motif linked by a MMP-2 substrate (GPLGVRG). (B) Schematic representation of the pET-29b(+) vector containing the MFK902 sequence. (C) Immunoblot of purified recombinant MFK902. (D) MMP-2–specific cleavage of MFK902 was performed as detailed in the Methods section.

The structure of βig-h3 consists of an RGD motif at the carboxyl terminal and four homologous fas-1 domains, each of which contains two highly conserved H1 and H2 sequences and one YH18 motif [34]. We previously developed the murine 4^th^ fas-1 peptide truncated for the H1 and H2 sequences, murine dhfas-1 (MFK00), to surmount the possibility of self-aggregation or structural changes induced by these segments [13]. To obtain improved therapeutic efficacy, we invented a MMP-2-cleavable peptide complex (MFK902), in which a MMP-2 substrate was inserted between the MFK00 and RGD peptides. The amino acid sequence of the MMP-2 substrate was GPLGVRG and matched the minimum sequence necessary for the active catalytic site [35] (Fig 1A). The scheme of MFK902 is shown in Fig 1B. Immunoblot analysis of purified MFK902 showed a 10.2-kD molecular weight in a single band, demonstrating its integrity and purity (Fig 1C).

To examine MMP-2-selectivity for MFK902 in the matrix-degrading enzymatic milieu of inflamed synovium, we treated *in vitro* MFK902 with a series of MMPs, including active MMP-1 (collagenase), MMP-2 (gelatinase), and MMP-3 (stromelysin), and cysteine proteases, such as cathepsin D, cathepsin L, and cathepsin K. MFK902 was subjected to concentration-dependent proteolytic cleavage by the active MMP-2. Cleavage of MFK902 started at a concentration of 200 ng/mL and reached its peak at 800 ng/mL. In contrast, there was no apparent cleavage of MFK902 by other active enzymes (Fig 1D).

### MFK902 Inhibited βig-h3-mediated Adhesion and Migration of Murine Fibroblasts

**Fig 2:**
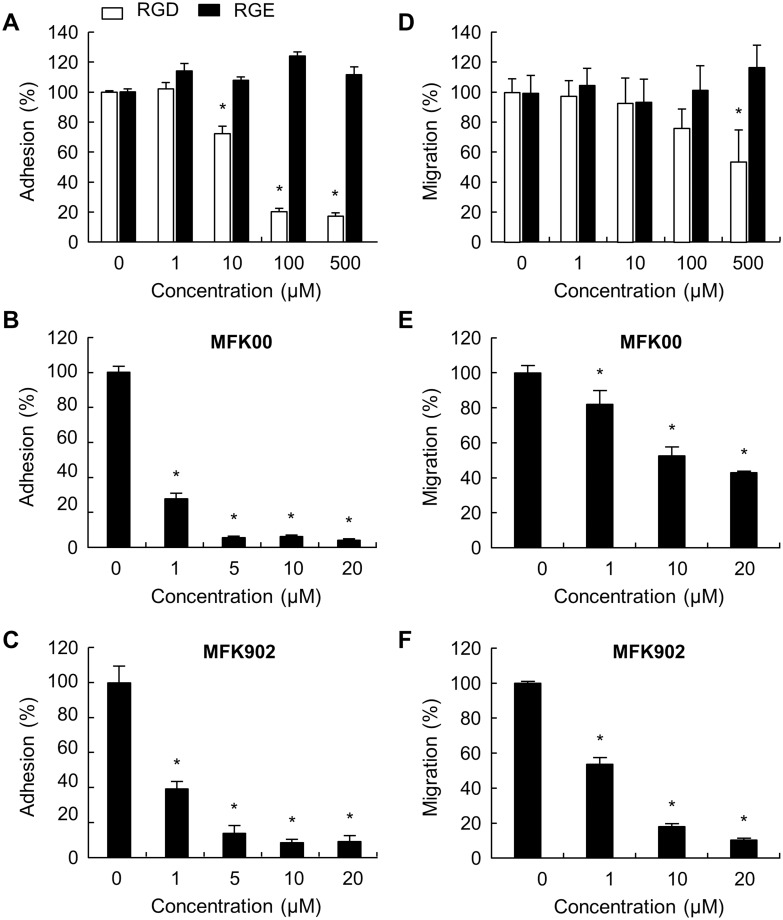
MFK902 inhibition of NIH3T3 cell adhesion and migration. βig-h3-derivatives, including RGD (GGRGDSP), MFK00, and MFK902 peptides, were prepared as described in the Methods section. Inhibition of adhesion by (A) the RGD and RGE, (B) MFK00, and (C) MFK902 peptides. Inhibition of migration by (D) the RGD and RGE, (E) MFK00, and (F) MFK902 peptides. *p < 0.05. Values are presented as the mean ± SEM.

Many of the cellular functions of fibroblastic cell subsets, including lung fibroblasts and FLS, are mediated by βig-h3 through interaction with integrins [6, 34]. The regulatory effect of MFK902 was validated by measurement of the adhesion and migration of murine fibroblast NIH3T3 cells on βig-h3-coated plates. The adhesion of fibroblasts on coated βig-h3 was weakly inhibited by the RGD peptide at a concentration of 10 μM, which showed a peak inhibition at 100 μM, and not inhibited at all by the RGE peptide (Fig 2A). Both MFK00 (Fig 2B) and MFK902 (Fig 2C) revealed a dose-dependent inhibition of βig-h3-mediated fibroblast adhesion and were similarly effective at lower concentrations (1 μM) relative to the RGD peptide.

In contrast to the adhesion study, the transwell migration assay indicated that the number of migrating cells to the lower surface of transwells coated with βig-h3 was significantly reduced at a very high concentration of RGD peptide (500 μM), which reduced migration by < 50% (Fig 2D). The lowest effective concentration of MFK00 for migration inhibition was 1 μM, with an IC_50_ of 13.56 ± 1.28 μM (Fig 2E). The IC_50_ of cell migration for MFK902 was 4.56 ± 0.25 μM, with the degree of inhibition > 2-fold at 1 μM and > 5-fold at 20 μM (Fig 2F). These data indicated that MFK902 was more potent than RGD and MFK00 peptides at inhibiting βig-h3-mediated functions *in vitro*.

### Treatment with MFK902 Attenuated Arthritis Severity in a Murine CIA Model

**Fig 3:**
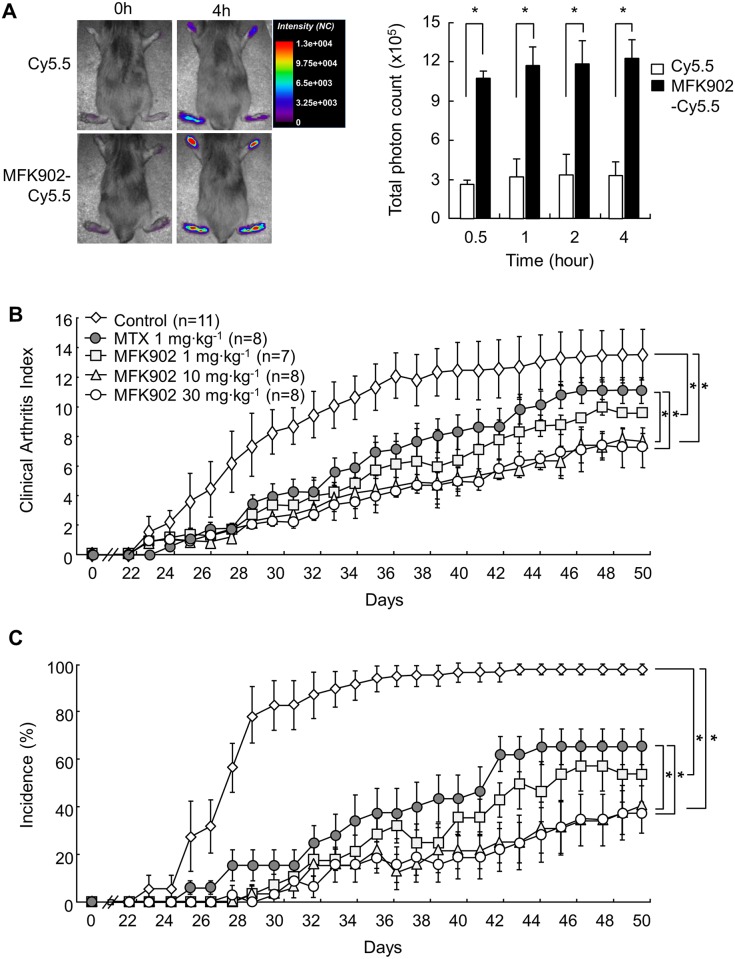
Modulation of clinical arthritis in mice with collagen-induced arthritis (CIA) after MFK902 treatment. (A) Distribution of Cy5.5-labeled MFK902 in the mice with active CIA. MFK902-Cy5.5 or free Cy5.5 were intravenously injected into mice and then near-infrared fluorescence (NIRF) images were captured using an eXplore Optix system. Representative color-coded images and total photon counts (mean at ± SD) indicated times are shown. (B-C) Mice with CIA were treated intraperitoneally with MFK902 daily at indicated doses, methotrexate (MTX, 1 mg/kg twice weekly), or phosphate-buffered saline (Control) beginning on day 23 after the first immunization. Arthritis scores are shown for 7–8 mice per group. (B) Clinical arthritis index. (C) Incidence of paw involvement. *p < 0.05. Data are expressed as the mean ± SEM.

To demonstrate whether MFK902 is accumulated in the inflamed joints, Cy5.5-labeled MFK902 and free Cy5.5 were intravenously injected to mice with CIA. MFK902 was four times more effectively accumulated in arthritic tissues compared with free Cy5.5 (TPCs 12.2 ± 1.4 x 10^5^ vs 3.3 ± 1.1 x 10^5^ for MFK902-Cy5.5 vs free Cy5.5 at 4h, respectively, *p* < 0.05) (Fig 3A).

We then determined whether MFK902 exhibited therapeutic effects on arthritis development in a murine CIA model that develops arthritis characterized by destructive chronic inflammation. Mice were treated intraperitoneally with three different dosages of MFK902 (1, 10, and 30 mg/kg) or vehicle daily, or with methotrexate (1 mg/kg) twice weekly, beginning at day 23 after the first immunization. The severity of arthritis was measured by CAI (Fig 3B) and incidence of inflamed paws (Fig 3C).

During the follow-up period, control mice showed a rapid progression of arthritis over the following 4 weeks. In contrast, all dosages of MFK902 resulted in a slower onset of arthritis, followed by a less steep increase in CAI up to day 50, with significantly lower scores (*p* < 0.05), compared with controls. Treatment with 10 and 30 mg/kg of MFK902 showed higher efficacy on modulation of arthritis activity, compared with methotrexate (*p* < 0.05). On day 50, the incidences of arthritic paws for the control and methotrexate groups were 94% and 65.9%, respectively, whereas those for the 1, 10, and 30 mg/kg groups with MFK902 treatment were 54%, 41%, and 38%, respectively (*p* < 0.05 for control vs. 1 mg/kg, vs. 10 mg/kg, or vs. 30 mg/kg, respectively) (Fig 3C). Taken together, these results pointed to a favourable therapeutic efficacy of MFK902 in a murine CIA model compared to methotrexate.

### Improvement of Pathologic Changes after MFK902 Treatment

**Fig 4:**
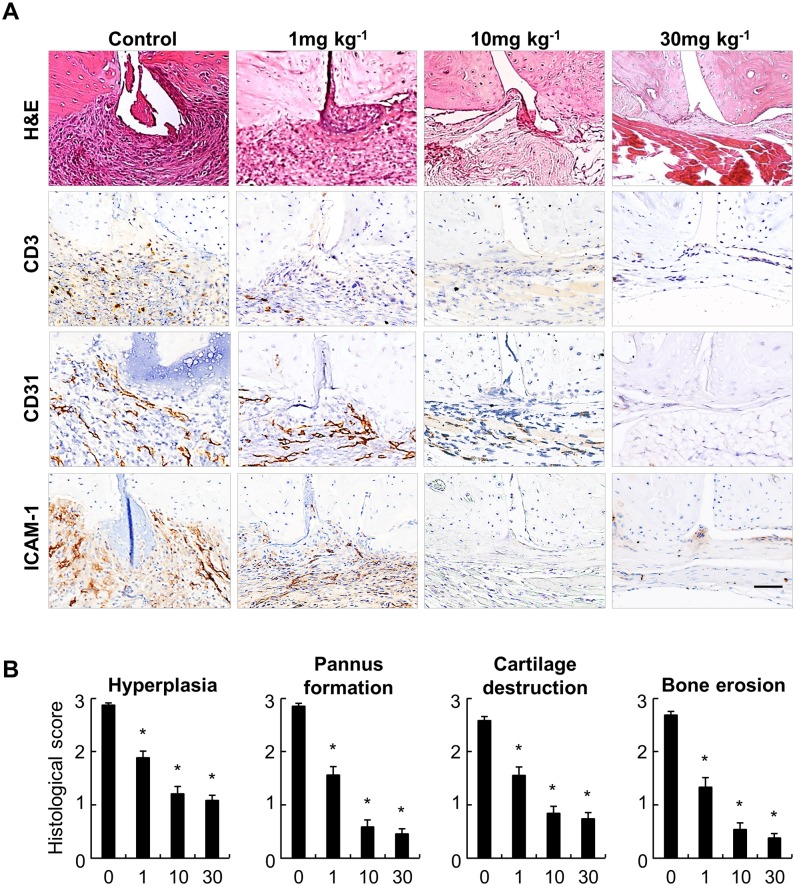
Amelioration of histopathologic severity in mice with CIA after MFK902 treatment. (A) Representative histopathologic findings of ankle joints from control and MFK902-treated mice on day 50. *Upper panel*, H&E-stained joints. *Lower panels*, Immunohistochemical staining for CD3, CD31, and ICAM-1 (brown). (B) Histologic scoring of arthritis severity in control and MFK902-treated mice (mg/kg). Histologic scores for synovial hyperplasia, pannus formation, cartilage destruction, and bone erosion (range: 0–3 for each parameter) were quantified as described in the Methods section. *p < 0.05 vs. controls. Data are expressed as the mean ± SEM. ICAM-1, intercellular adhesion molecule-1. Scale bar: 50 μm

To test whether the clinical improvement was accompanied by histopathologic changes, the structural integrity of synovial tissues from CIA mice was analysed (Fig 4A). Histology of joint tissues from the control mice revealed profound synovial hyperplasia, inflammatory cell infiltration, and pannus formation, as well as highly evident cartilage destruction and bone erosion. In contrast, all these pathologic findings were markedly inhibited by treatment with 10 mg/kg of MFK902, while they were attenuated moderately by treatment with 1 mg/kg of MFK902.

We then performed immunohistochemical staining to examine inflammatory processes in synovial tissues (Fig 4A). Infiltration of T cells within the sublining layer was reduced after treatment with MFK902 in a dose-dependent manner up to 10 mg/kg. Furthermore, changes in angiogenesis and ICAM-1 expression showed similar patterns after MFK902 treatment.

To compare the histologic disturbances quantitatively among these groups, we measured the histologic scores using an arbitrary scale from 0 to 3 for synovial hyperplasia, pannus formation, cartilage destruction, and bone erosion (Fig 4B). Histologic analysis of inflamed joint tissues corroborated the clinical data of arthritis. Compared with that of the control group, treatment even with the lowest dosage (1 mg/kg) significantly reduced histologic scores, which were further suppressed by treatment with higher dosages (10 and 30 mg/kg). Taken together, these results reflected that MFK902 ameliorated arthritis through attenuation of pathologic disorganization.

### Modulation of Inflammatory Mediators by MFK902 Treatment

**Fig 5:**
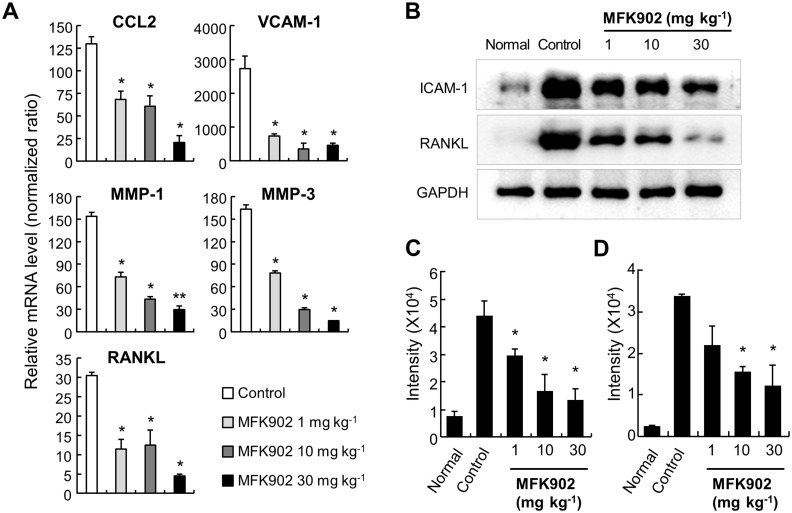
Downregulation of inflammatory mediators after MFK902 treatment in the arthritis tissues from CIA mice. (A) Semi-quantitative measurement of inflammatory mediators from joint tissues using reverse transcription-polymerase chain reaction (RT-PCR). The comparison of transcript levels was performed using 18S ribosomal RNA as the denominator and normalized according to the relative expression level of inflammatory mediators from non-arthritic mice. (B) Immunoblot analysis of ICAM-1 and RANKL expression. *p < 0.05 vs. controls. Values are expressed as the mean ± SEM. CCL2, chemokine (C-C motif) ligand 2; ICAM-1, intercellular adhesion molecule-1; RANKL, Receptor activator of nuclear factor kappa-B ligand; VCAM-1, vascular cell adhesion molecule-1.

To further validate the mechanism by which MFK902 suppressed arthritis progression, we examined the transcript expression of chemokines (CCL2), adhesion molecules (VCAM-1), MMPs (MMP-1 and -3), and RANKL by semi-quantitative RT-PCR (Fig 5A). MFK902 significantly downregulated the expression of these inflammatory mediators in parallel with improved clinical features and pathologic parameters as compared with controls.

We then quantified inflammatory mediators isolated from inflamed joint tissues at the protein level (Fig 5B). Immunoblot assays using whole extracts of inflamed paws from CIA mice revealed a dose-dependent reduction in ICAM-1 levels following MFK902 treatment. Quantification of blot images using ImageJ software revealed significant decreases in ICAM-1 at all dosages of MFK902 (Fig 5B), which was consistent with attenuated ICAM-1 expression observed from the immunohistochemical stain. Protein levels of RNAKL were significantly reduced in mice treated with higher dosages of MFK902 (10 and 30 mg/kg) as compared to controls, which supported the underlying pathophysiologic mechanism of ameliorated bone erosions in treated mice.

### MFK902 Safety and Tolerability

Toxicity and intolerance impede preclinical drug development, despite the drugs showing remarkable efficacy. To test the safety of MFK902 *in vivo*, we first evaluated the adverse effects on hematologic parameters (Table 1). MFK902 did not influence white blood cell count, haemoglobin level, or platelet count in peripheral blood. We detected no deleterious effects on the liver function, as reflected by levels of aspartate transaminase and alanine transaminase, or on renal function, based on tests, including blood urea nitrogen and creatinine. Moreover, there were no alarming changes in the body weight of MFK902-treated mice. These data indicated that MFK902 was safe and tolerable for the treatment of chronic inflammatory arthritis.

**Table 1:** *In vivo* safety profile in CIA mice treated with MFK902.

Group	Body weight(Δg)	RBC(×10^6^/μL)	WBC(×10^3^/μL)	Platelet(×10^3^/μL)	AST(U/L)	ALT(U/L)	BUN(mg/L)	Creatinine(mg/L)
Control	2.6 ± 0.0	11.4 ± 0.8	5.3 ± 2.7	1062.6 ± 261.6	75.0 ± 6.0	51.0 ± 11.0	220.0 ± 45.0	5.0 ± 0.4
1 mg/kg	1.0 ± 0.8	11.0 ± 0.9	3.4 ± 1.0	967.7 ± 37.0	115.0 ± 14.0	58.0 ± 1.1	300.0 ± 4.0	6.0 ± 0.0
10 mg/kg	1.0 ± 0.7	11.5 ± 0.5	3.6 ± 0.1	1240.0 ± 76.0	72.0 ± 9.2	34.0 ± 3.2	270.0 ± 4.0	5.0 ± 0.1
30 mg/kg	1.0 ± 0.9	13.0 ± 0.5	2.1 ± 1.1	1062.0 ± 7.0	161.0 ± 10.0	49.0 ± 10.0	190.0 ± 0.0	4.0 ± 1.0

Data are presented as the mean ± SEM. AST, aspartate transaminase; ALT, alanine transaminase; BUN, blood urea nitrogen.

## Discussion

Our goal was to develop a MMP-2-cleavable integrin-dispersing prodrug that would target the surface of resident effector cells haptotactic to ECM proteins within the inflammatory microenvironment of chronic inflammatory arthritis. In this study, we demonstrated that a MMP-2-cleavable peptide complex based on the βig-h3 structure (MFK902) potently restrained existing chronic inflammatory arthritis. MFK902 decomposed specifically in the presence of active MMP-2 and inhibited βig-h3-mediated adhesion and migration of murine fibroblasts more effectively as compared to MFK00 or RGD peptides. Treatment with MFK902 efficiently modulated arthritis activity in a CIA model, which occurred in parallel with improved histologic deterioration and reduction of inflammatory mediator expression in joint tissues. In addition, we observed a favourable safety profile associated with MFK902 treatment *in vivo*, which indicated that MFK902 represents a potentially effective approach to RA treatment.

Exploiting pathophysiological alterations in cells, such as FLS involved in pannus formation, or the extracellular microenvironment, including adhesion molecules, ECM proteins, and matrix proteinases within inflamed tissues, has been an encouraging method involving tissue-targeted drug delivery [13, 16, 36–39]. MMPs are used in MMP-activated drug delivery, such as MMP-activated peptide prodrugs, MMP-activated carrier-peptide drug conjugates, MMP-activated DNA delivery, and MMP-deprotection of liposomes, in which MMPs cleave prodrugs or drug-delivery systems, followed by the selective release of activated drugs in targeted tissues overexpressing MMPs [16]. We previously developed a novel MMP-exploiting prodrug, which was designed as a MMP-cleavable composite peptide with two different effectors linked by an MMP-specific substrate. Here, we focused on MMPs anchored to cell surface, which may potentiate pericellular focalization of active drugs after cleavage by anchored MMPs.

MMP-2 is an MMP typically anchored on the cell surface following secretion in order to target their substrates in the surrounding cellular environment [16]. Compartmentalized MMP-2 utilizes its C-terminal haemopexin-like domain for MMP-2-αvβ3 integrin-complex formation in a RGD-independent manner [24, 40] and utilizes its collagen-binding domain for MMP-2-type I collagen-β1-integrin complex formation [41]. Based on these mechanisms, dual-targeted radiolabelled probes [42, 43] and chemotherapeutics [43] that bind to the αvβ3 integrin and are then activated by MMP-2 have been developed. MMP-2 is one of the overexpressed MMPs in the rheumatoid synovium [1, 18, 19] and highly expressed on the cell surface of synoviocytes, endothelial cells, and infiltrating monocytes and macrophages, especially in the pannus [18, 19]. Therefore, taking advantage of increased MMP-2 expression on the cell surface within arthritis tissues may constitute a reasonable approach to MMP-2-mediated therapeutics.

Recently, βig-h3-based therapeutic agents for inflammatory diseases have attracted increased attention [13–15]. The potential of βig-h3 derivatives as RA therapies focused on the interruption of βig-h3-mediated FLS function [6, 13]. We previously developed a modified fas-1 peptide (MFK00) and peptide complexes consisting of MFK00 and RGD peptides either linked by a MMP-1 substrate (MFK24) or not as RA therapeutic agents and validated their regulatory role on βig-h3-mediated cellular functions *in vitro* and their therapeutic efficacy on chronic arthritis using a murine CIA model [13]. MFK24 most effectively suppressed βig-h3-mediated fibroblast adhesion and migration and promoted the efficient accumulation and conspicuous amelioration of arthritis *in vivo*. These results indicate that this proof-of-concept design of MMP-cleavable peptides is a platform technology expandable for RA therapy.

In this study, we designed a novel peptide complex (MFK902) that positioned a MMP-2 substrate between RGD and MFK00 motifs. This concept was based on the hypothesis that abundantly expressed MMP-2 on both the cell surface and the ECM space within inflamed joints [1, 18, 19] would efficiently cleave MFK902. We then hypothesized that MFK902 delivered to inflamed synovium would bind to αvβ3 integrin through the presence of either the MFK00 or RGD peptides. Based on a previous report indicating that the binding reaction of βig-h3 and αvβ3 integrin involves a 1:1 stoichiometry despite of the presence of several αvβ3 integrin-binding motifs on βig-h3 [11], we speculated that MFK902 containing dual-interacting motifs would bridge two integrins positioned back to back and then be split into an individual motif-integrin complex by active MMP-2 bound to αvβ3 integrin on the FLS. This cleavage may inhibit integrin clustering, which is critical for activation of intracellular signalling pathways [44] that regulate cellular functions, such as adhesion, migration, proliferation, and survival.

Our assumption was successfully demonstrated by the therapeutic efficacy displayed by MFK902 on a chronic inflammatory arthritis model, and was further supported by the improvement in pathologic changes and decreased expression of inflammatory mediators within arthritic tissues. However, our study had a few limitations. First, there were potential mechanisms not addressed in the present study. MMP-2 expression is upregulated in endothelial cells, as well as FLS, within the rheumatoid synovium [18, 19]. Moreover, the fas-1 domain from βig-h3 is an important endogenous regulator of pathologic angiogenesis [15]. Therefore, another mechanism of action may involve modulation of abnormal angiogenesis within inflamed synovium, which may coordinate with suppression of FLS functions and ameliorate chronic inflammatory arthritis. Second, we did not make direct comparisons of the *in vitro* and *in vivo* efficacies between MMP-1- and MMP2-cleavable peptide complexes. Although the primary purpose was to test this novel proof-of-concept technology of utilizing MMP-2, which is expressed on the cell surface but at a relatively small amount, as the cleaving armament, positive results of this study warrant further studies including a direct comparison of efficacy.

In summary, we demonstrated that MFK902 was a potent and safe prodrug capable of exploiting overexpressed MMP-2 within inflamed synovium. Our *in vivo* arthritis model demonstrated a clear therapeutic benefit derived from systemic administration of a MMP-2-cleavable peptide complex to reduce the inflammatory cascades associated with destructive arthritis. These data supported the future study of this promising therapeutic platform for treatment of chronic inflammatory arthritis, as well as the exploitation of novel strategies that target specific matrix proteinases in the study of inflammatory synovitis.

## Abbreviations

βig-h3: Transforming growth factor-β-inducible gene-h3
CAI: clinical arthritis index
CCL2: chemokine (C-C motif) ligand 2
CIA: collagen-induced arthritis
ECM: extracellular matrix
FLS: fibroblast-like synoviocytes
ICAM-1: intercellular adhesion molecule-1
MMP: matrix metalloproteinase
MMP-2: matrix metalloproteinase-2
RA: rheumatoid arthritis
RANKL: Receptor activator of nuclear factor kappa-B ligand
VCAM-1: vascular cell adhesion molecule-1
